# Nutritional status and quality of life in interstitial lung disease: a prospective cohort study

**DOI:** 10.1186/s12890-021-01418-5

**Published:** 2021-02-05

**Authors:** Alisar A. Kanjrawi, Lara Mathers, Susanne Webster, Tamera J. Corte, Sharon Carey

**Affiliations:** 1 Nutrition and Dietetics, University of Sydney, Sydney, NSW 2006 Australia; 2 Department of Nutrition and Dietetics, Royal Prince Alfred Hospital, Missenden Road, Camperdown, NSW 2050 Australia; 3 Department of Respiratory Medicine, Royal Prince Alfred Hospital, Missenden Road, Camperdown, NSW 2050 Australia; 4 Sydney Medical School, University of Sydney, Sydney, NSW 2006 Australia; 5 Centre of Research Excellence for Pulmonary Fibrosis, Royal Prince Alfred Hospital, Camperdown, NSW 2050 Australia

**Keywords:** Lung disease, Nutritional status, Quality of life

## Abstract

**Background:**

Malnutrition and altered body composition are well-documented in chronic pulmonary diseases; however, investigation of nutritional status in interstitial lung disease (ILD) is limited. This study aimed to describe the nutritional status of ILD patients within three diagnostic groups and explore the relationship between nutritional status and quality of life (QoL).

**Methods:**

Consecutive patients attending an ILD clinic within a tertiary referral hospital in Sydney, Australia were studied. Weight, body-mass-index, anthropometrics, handgrip strength (HGS), subjective global assessment and QoL questionnaires (EQ-5D-5L and King’s-Brief Interstitial-Lung-Disease ‘K-BILD’) were collected. Associations between nutritional status and QoL were analysed.

**Results:**

Ninety participants were recruited and categorised: (1) Idiopathic Pulmonary Fibrosis (IPF) (2) Connective-Tissue Disease associated-ILD (CTD-ILD) or (3) Other (non-IPF/non-CTD ILD). Median age was 66.5 (18) years. Four-percent of patients were underweight and 50% were overweight or obese. Median HGS was 71%-(25.3) of predicted and was correlated to all measures of QoL including EQ-5D health-state index (r = 0.376, *p* < 0.0001), patient-reported EQ-5D-5L Visual Analogue Score (r = 0.367, *p* < 0.0001) and K-BILD total score (r = 0.346, *p* = 0.001). Twenty-three percent of the variance in K-BILD total score (F = 12.888, *p* < 0.0001) was explained by HGS (ß = 0.273, *p* = 0.006) and forced vital capacity % predicted (ß = 0.331, *p* = 0.001).

**Conclusions:**

Although a small number of ILD patients were malnourished, a large proportion of the cohort were overweight or obese. Handgrip strength was compromised and correlated to QoL. Future research with a larger cohort is required to explore the role of HGS as a predictor of QoL.

## Background

Interstitial lung diseases (ILD) are a heterogeneous group of chronic lung disorders that share common clinical features of inflammation and/or scarring of the lung interstitium [1]. ILD can be associated with exposure to certain medications, toxins and pollutants including silica dust and asbestos fibres or secondary to connective tissue diseases such as rheumatoid arthritis and systemic sclerosis [2]. The diagnosis of ILD can be categorised into three categories, the first of which is idiopathic pulmonary fibrosis (IPF). Idiopathic pulmonary fibrosis (IPF) is a relentlessly progressive and fatal disorder, which has a median survival rate of 2–5 years [3], is the most common form of idiopathic interstitial pneumonia [4]. Secondly, connective tissue disease associated ILD (CTD-ILD) which is considered one of the most common subtypes of ILD of known aetiology [5]. And thirdly, ILD which is unable to be categorised as above, termed idiopathic interstitial pneumonias (non-IPF/non-CTD). Overall, ILD patients have reduced quality of life, substantial morbidity and early mortality [6, 7]. Patients often experience a range of disabling symptoms including dyspnoea and cough as well as non-respiratory symptoms such as depression, anxiety and fatigue and weight loss, all of which may contribute to the worse health related quality of life reported in ILD patients [8, 9].

The role of diet and nutrition in the progression and prognosis of respiratory disease is increasingly recognised in the literature. Nutrition in chronic lung diseases is complex as both obesity as well as malnutrition exist [10]. Malnutrition is well-documented in pulmonary diseases such as chronic obstructive pulmonary disease (COPD) and cystic fibrosis (CF) due to inadequate oral intake related to the prevalence of multiple symptoms such as anorexia and reflux, as well as increased metabolic requirements [11]. Similar symptoms that affect oral intake and increase energy requirements are also prevalent in ILD. Malnutrition in COPD, resulting in low body mass index (BMI) and fat free mass (FFM), serves as a poor prognostic factor and is associated with higher mortality [12]. Obesity is also prevalent in chronic lung diseases and can have detrimental effects [10]. However, research has shown that higher BMI is associated with longer survival and lower mortality in COPD [13], while underweight patients have higher mortality risk and increased occurrence of acute exacerbations. Similarly, under nutrition is linked to poor clinical outcomes in CF patients [14].

Recently, it has been suggested that lower body mass index and weight loss are both associated with increased mortality in ILD patients [15]. In the present study, we hypothesised that malnutrition, weight loss and decreased BMI is prevalent in interstitial lung disease and this may affect quality of life. The purpose of this study was to describe the nutritional status of ILD patients within three pre-specified diagnostic groups and explore the relationship between nutritional status and quality of life.

## Methods

### Study design and measures

In this prospective, cross-sectional cohort study, consecutive adult patients attending the Royal Prince Alfred Hospital ILD Clinic between 21st August 2019 and 10th October 2019 were contacted prior to their appointment or approached during their visit to the clinic and prospectively invited to participate in the study. Participants gave informed written consent upon agreement to participate. Patients were excluded if they had not yet received an ILD diagnosis, were unable to consent due to cognitive impairment, or were non-English speaking without an interpreter. The present study was approved by the Royal Prince Alfred Ethics committee (Protocol No. X16-0398, HREC/16/RPAH/569).

### Nutritional status

Nutritional status was assessed by the following items:

1. Weight and height were obtained from medical records (most recent Lung Function Report within previous 3 months), which were measured using calibrated Seca 286 ultrasonic measuring station scale and recorded to nearest 0.1 kg and 0.1 cm respectively on the index visit date. Participants were in light day clothing with shoes off. BMI was calculated using standard formula weight (kg)/height (m^2^), and healthy weight range was ≥ 18.5 to < 25 kg/m^2^ for people aged less than 65 and ≥ 22 to < 30 kg/m^2^ for people 65 years of age and over [16, 17].
2. Anthropometric measures were taken by student dietitian including mid-arm circumference (MAC) and triceps skinfold (TSF). Mid-arm muscle circumference (MAMC), a marker of lean muscle mass, was calculated from MAC and TSF using standard formula MAMC = MAC − (3.1415 × TSF). All anthropometry was performed on the non-dominant arm, technique in accordance with recommended practice. Equipment included a calibrated John Bull Harpenden Skinfold Caliper (British Indicators Ltd, Weybridge, UK) and a standard measuring tape. Standard percentile ranges for age and gender were used for comparison of MAC, MAMC and TSF.
3. Muscle strength was measured using handgrip strength (HGS) with a calibrated North Coast Hydraulic Hand Dynamometer (North Coast Medical Inc, Morgan Hill, USA). HGS was measured using the non-dominant arm while participants were seated and the arm was at a right angle. The average of three consecutive measures were taken and percentage of predicted HGS was calculated using standard formula [18].
4. Subjective Global Assessment (SGA), is a validated tool and a component of nutritional assessment that uses subjective measures to rank the severity of malnutrition. It takes into consideration weight, weight change, dietary intake and change, duration and frequency of gastrointestinal symptoms and functionality. It also includes a physical examination of sites related to subcutaneous fat and muscle stores. Patients are categorised as SGA (A) ‘well nourished’, SGA (B) ‘mild-moderate malnutrition’ and SGA (C) ‘severe malnutrition’ [19].

### Quality of life measures

Quality of life was assessed using two self-administered questionnaires. Firstly, the EuroQol Group ‘EQ-5D-5L’Version 1, is a widely used generic measure of health status which consists of two parts, a descriptive system and a Visual Analogue Scale (VAS). The descriptive system examines 5 dimensions; mobility, self-care, usual activities, pain/discomfort and anxiety/depression via a five-point Likert-Scale. These scores are combined using a distinct algorithm to create a health ‘utility’ index ranging from − 0.208 which is considered the worst possible health state up to a value of 1, indicating the best possible health state. In this study, health state index scores were calculated using crosswalk value sets for the UK. The Visual Analogue Scale allows participants to value current health on a scale between 0 and 100, where higher values represent better health [20].

Secondly, the ‘King’s Brief Interstitial Lung Disease’ (K-BILD) questionnaire, which is an ILD-specific health-related quality of life questionnaire that measures health impairment induced by ILD. It comprises 15 items and a seven-point Likert response scale. It has three domains: ‘breathlessness and activities’, ‘psychological impact’, and ‘chest symptoms’ [21]. Total and domain-specific scores range from 0 to 100, with higher values indicating better health [22].

### Clinical and lung function data

Medical records were reviewed to obtain primary diagnosis, which was used to categorise participants into one of the following diagnostic groups: (1) Idiopathic pulmonary fibrosis (IPF), (2) connective tissue disease (CTD) associated ILD (CTD-ILD) and (3) other (non IPF/non-CTD ILD).The following subtypes of ILD were included in the CTD-ILD group: Rheumatoid Arthritis, Systemic Lupus Erythematosus, Systemic Sclerosis, Dermatopolymyositis, Polymyositis, Vasculitis, Antisynthetase Syndrome, Scleroderma, Idiopathic Pneumonia with Autoimmune Features, undifferentiated Connective Tissue Disease and mixed Connective Tissue disease. The other (non-IPF/non-CTD ILD) group included participants with the following diagnoses: hypersensitivity pneumonitis, sarcoid and unclassifiable ILD. Other data collected from medical records included age, gender, ethnicity, smoking status, diagnosis of pulmonary hypertension, medications (corticosteroids, immunosuppression, anti-fibrotic agents, anti-reflux therapy and oxygen therapy). Lung function parameters (forced vital capacity ‘FVC’, forced vital capacity %predicted ‘FVC%’, diffusing capacity of the lungs for carbon monoxide %predicted ‘DLCO%’, total lung capacity ‘TLC’, total lung capacity %predicted ‘TLC%’) were also collected from the most recent Lung Function Report in medical records (within previous 3 months).

### Statistical analysis

Data was collected on a pre-designed data collection sheet de-identified and entered into and analysed using the Statistical Package for Social Sciences version 25.0 (SPSS Inc., Chicago, IL). Differences in nutritional status across the three diagnostic groups were examined using Kruskal–Wallis and Chi-square tests. Pearson’s correlation coefficient and multiple linear regression were used to determine associations between nutritional status and quality of life. Data for non-participants was collected from electronic medical records including age, gender, ethnicity, weight, BMI and diagnosis; and compared to that of participants using Kruskal–Wallis and Chi-square tests to ensure no selection bias. Statistical significance of *p* < 0.05 was assumed.

## Results

### Patient characteristics

In total, 164 out of 220 patients screened met the inclusion criteria. Of the 99 consecutive patients approached (65 were not approached due to limited resources), only 9 declined to participate. Thirty-seven males and 53 females consented to participate, with a final study cohort of 90 participants and a participation rate of 91%. There was no significant difference between participants and non-participants. Participant characteristics are summarised in Table 1. Median age was 66.5 (18) years and the majority Caucasian (84.4%) with 58.9% being female. Interestingly, however, there were more males (70.4%) in the IPF group (*p* = 0.01). Participants with CTD-ILD were younger than in the IPF and ‘non IPF/non CTD-ILD’ groups (*p* < 0.0001), and there were more smokers in the IPF group (*p* < 0.0001). There were no significant differences among the three diagnostic groups in terms of ethnicity, presence of pulmonary hypertension, lung function parameters, use of continuous supplemental oxygen and anti-reflux therapy. Most participants with IPF were treated with anti-fibrotic agents, while those with CTD-ILD were often prescribed corticosteroids and immunosuppressant medication.

**Table 1:** Basic demographics and medical information

	Total	IPF	CTD-ILD	Non-IPF/non-CTD
	*n* = 90	*n* = 27 (30%)	*n* = 34 (37.8%)	*n* = 29 (32.2%)
Female^a,b^	53 (58.9%)	8 (29.6%)	24 (70.6%)	21 (72.4%)
Age^a,c^ (years)	66.5 (18)	71.0 (12)	56.0 (16)	68.0 (15)
Ethnicity				
Caucasian	76 (84.4%)	25 (92.6%)	25 (73.5%)	26 (89.7%)
Other	14 (15.6%)	2 (7.4%)	9 (26.5%)	3 (10.3%)
Smoking Status^a,b^				
Never smoked	44 (48.9%)	5 (18.5%)	19 (55.9%)	20 (69.0%)
Ever smoked	46 (51.1%)	22 (81.4%)	15 (454.1%)	9 (31.0%)
Treatments				
Cortico-steroids^a,b^	43 (47.8%)	2 (7.4%)	22 (64.7%)	19 (65.5%)
Anti-fibrotic agents^a,b^	26 (28.9%)	24 (88.9%)	1 (2.9%)	1 (3.4%)
Immunosuppression^a,b,c^	35 (38.9%)	0	25 (73.5%)	10 (34.5%)
Anti-reflux therapy	30 (33.3%)	9 (33.3%)	12 (35.3%)	9 (31.0%)
Supplemental oxygen				
Continuous	11 (12.2%)	5 (18.5%)	3 (8.8%)	3 (10.3%)
Pulmonary hypertension				
Present	12 (13.3%)	2 (7.4%)	5 (14.7%)	5 (17.2%)
Lung function				
FVC %Predicted *n* = 90	74 (31)	77 (45)	74 (32)	56 (47)
DLCO %Predicted *n* = 88	51 (26)	47.5 (23)	49 (46)	51 (33)
TLC %Predicted *n* = 40	66 (27)	63 (39)	67 (27)	66 (29)

All data shown as number (percent) or median (interquartile range)

*IPF* idiopathic pulmonary fibrosis, *CTD-ILD* connective-tissue disease associated interstitial lung disease, *FVC* forced vital capacity, *DLCO* diffusing capacity of the lungs for carbon monoxide, *TLC* total lung capacity

^a^Significant difference between IPF and CTD-ILD

^b^Significant difference between IPF and other

^c^Significant difference between CTD-ILD and non-IPF/non-CTD

### Overall nutritional status

Nutritional status measures can be seen in Table 2. Mild-moderate malnutrition (SGA B) was identified in 8.9% of participants. The majority of malnourished participants belonged to the IPF group (*p* = 0.041) and all were above 65 years of age. Median BMI was 28.3 (7.7) kg/m^2^, with only 4.4% of patients in the underweight range, 45.6% in the healthy range and 50% in the overweight and obese range. Median triceps skinfolds, mid-arm circumference and mid-arm muscle circumference fell between the 75th–90th percentile, 50th–75th percentile and 25th–50th percentile, respectively. Median handgrip strength was 71% (25.3) of predicted and decreased significantly with increasing age (r = − 0.211, *p* = 0.046). There was no statistically significant difference between the three diagnostic groups in terms of weight, BMI, %predicted handgrip strength, TSF percentile, MAC percentile and MAMC percentile (Table 2).

**Table 2:** Nutritional status measures across the diagnostic groups

	Total	IPF	CTD-ILD	Non-IPF/non-CTD
	(*n* = 90)	(*n* = 27)	(*n* = 34)	(*n* = 29)
Weight (kg)	77.8 (27.5)	76.7 (20.6)	75.4 (27.3)	78.5 (29.8)
BMI (kg/m^2^)	28.3 (7.7)	26.8 (5.6)	28.7 (8.7)	30.6 (9.7)
Underweight	4 (4.4%)	1 (3.7%)	1 (2.9%)	2 (6.9%)
Healthy weight	41 (45.6%)	20 (74.1%)	12 (35.3%)	9 (31.0%)
Overweight/obese	45 (50%)	6 (22.2%)	21 (61.8%)	18 (62.0%)
SGA				
A	82 (91.1%)	22 (81.5%)	34 (100.0%)	26 (89.7%)
B	8 (8.9%)	5 (18.5%)	0 (0.0%)	3 (10.3%)
C	0	0	0	0
Grip strength (kg)	22 (15.9)	27.0 (16.3)	19.2 (17.7)	19.7 (10.6)
% Predicted grip strength	71.0 (25.3)	74.7 (20.5)	70.0 (28.7)	70.3 (28.4)
TSF^a,b^ (mm)	22.0 (15.1)	19.5 (7.6)	23.8 (12.9)	24.5 (16.8)
TSF percentile	75th–90th	50th–75th	50th–75th	75th–90th
Data missing	8	0	2	6
MAC (cm)	30 (4.3)	29.6 (3.4)	31.8 (4.5)	30.8 (6.5)
MAC percentile	50th–75th	25th–50th	50th–75th	50th–75th
Data missing	6	0	1	5
MAMC (cm)	23.7 (4.1)	24.3 (5.6)	23.7 (3.8)	23.3 (5.0)
MAMC percentile	25th–50th	25th–50th	50th–75th	10th–25th
Data missing	8	0	2	6
EQ-5D-5L				
Index Value (− 0.594 to 1)	0.727 (0.232)	0.762 (0.201)	0.725 (0.210)	0.683 (0.286)
VAS^b,c^ (0–100)	70.0 (25.0)	75.0 (20.0)	77.5 (26.0)	60.0 (33.0)
K-BILD (0–100)				
Total score	66.7 (17.5)	70 (32.2)	67.2 (33.3)	61.7 (32.2)
Breathlessness and activity	45.8 (45.8)	45.8 (45.8)	50.0 (43.8)	35.4 (46.9)
Chest symptoms	77.8 (27.8)	77.8 (27.8)	77.8 (27.8)	77.8 (48.6)
Psychological	71.4 (39.3)	73.8 (40.5)	72.6 (32.1)	63.1 (35.1)

Data shown as number (percent) or median (interquartile range)

*IPF* idiopathic pulmonary fibrosis, *CTD-ILD* connective-tissue disease associated interstitial lung disease, *BMI* body mass index, *SGA* subjective global assessment, *TSF* triceps skinfold, *MAC* mid-arm circumference, *MAMC* mid-arm muscle circumference

^a^Significant difference between IPF and CTD-ILD

^b^Significant difference between IPF and non-IPF/non-CTD

^c^Significant difference between CTD-ILD and non-IPF/non-CTD

### Quality of life

The two QoL questionnaires K-BILD and EQ-5D-5L were strongly correlated (r = 0.637, *p* < 0.0001). QoL was not correlated to age, weight or BMI.

Those who were found to have ‘mild-moderate’ malnutrition scored significantly lower on K-BILD’s breathlessness and activity domain (*p* = 0.021). However, no significant differences were found between well-nourished and malnourished patients in EQ-5D health state index scores and K-BILD total, psychological and chest symptoms domains scores. Handgrip strength was significantly correlated to all measures of QoL including EQ-5D health state index scores (r = 0.376, *p* < 0.0001), patient-reported EQ-VAS (r = 0.367, *p* < 0.0001) and K-BILD total score (r = 0.346, *p* = 0.001) (Table 3).

**Table 3:** Quality of life measures across nutritional groups as defined by SGA

	Total	SGA A	SGA B
EQ-5D-5L			
Health Index (− 0.594 to 1)	0.727 (0.232)	0.732 (0.226)	0.639 (0.349)
VAS (0–100)	70.0 (25.0)	75.0 (21.0)	60.0 (34.0)
K-BILD (0–100)			
Total score	66.7 (17.5)	67.8 (31.7)	46.1 (35.0)
Breathlessness and activity^a^	45.8 (45.8)	45.8 (43.8)	25.0 (33.3)
Chest symptoms	77.8 (27.8)	77.8 (27.8)	61.1 (45.8)
Psychological	71.4 (39.3)	71.4 (36. 9)	61.9 (39.3)

Data shown as median (interquartile range)

*SGA* subjective global assessment, *VAS* Visual Analogue Scale, K-BILD: King’s Brief Interstitial Lung Disease questionnaire

^a^Significant difference between SGA A and SGA B

A stepwise backward linear regression model was used to assess which measures might explain quality of life for this patient group. Weight, BMI, %predicted handgrip strength and age were included in the analysis, along with lung function parameters. In the final model, 23.1% of the variance in K-BILD total score (F = 12.888, *p* < 0.0001) was explained by handgrip strength (ß = 0.273, *p* = 0.006) and forced vital capacity %predicted (ß = 0.331, *p* = 0.001). Handgrip strength alone (ß = 0.402, *p* < 0.0001) accounted for 16.2% of variability in EQ-5D health state index scores (F = 16.985, *p* < 0.0001).

## Discussion

The present study aimed to assess the nutritional status of ILD patients and compare it across three pre-specified diagnostic groups, as well as explore the relationship between nutritional status and quality of life. Unexpectedly, findings show that malnutrition and decreased BMI are uncommon amongst this cohort of patients with ILD. Malnutrition was identified in a small number of participants, mainly in the older IPF group, however the majority of participants were found to be well-nourished according to SGA assessment. Nevertheless, handgrip strength was compromised in all subgroups compared to normative values. As for group comparisons, no significant differences were found in weight, BMI and anthropometric measures; triceps skin fold, mid-arm circumference and mid-arm muscle circumference across the three diagnostic groups. While nutritional status was not shown to be strongly correlated to quality of life, there was a trend towards lower quality of life with compromised nutritional status, and a significant impact on breathlessness and activity scores in the K-BILD for malnourished compared to well-nourished participants. Lower handgrip strength was also associated with reduced quality of life.

While a small number of participants were found to be underweight, half the study cohort was found to be overweight or obese. Obesity was predominantly found in the CTD-ILD and non-IPF/non-CTD groups which may indicate differing aetiology impacting the nutritional journey of the patient. Alternatively this tendency towards a larger BMI may be representative of a relatively well cohort which is consistent with measures of lung functionality. The nutritional profile of the IPF participants in this cohort fit within the healthy weight range with some undernutrition. This finding is similar to previous literature assessing nutritional status in IPF.

Alakhras et al. reported adiverse range from underweight to obesity in an IPF population [23]. Research shows that both being underweight and overweight has an impact on lung function. Patients with greater weight and lean mass losses, have greater deterioration in lung function, while obesity complicates breathing and leads to decreased performance and increased workload [24, 25].

There are conflicting results regarding the relationship between weight status with disease outcomes and survival in chronic lung diseases. For example, in COPD data from the COPDGene study suggests that obesity is linked to worse dyspnoea, increased risk of acute exacerbations and poorer quality of life [26]. On the contrary, other studies suggest that obese patients in fact have lower acute exacerbation frequency [27]. The study by Landbo et al. investigated the relationship between weight status, disease severity and survival, in mild to moderate COPD a normal body weight or being overweight was favourable for improved prognosis. In end stage (severe) COPD, being overweight or obese was actually associated with better survival [28]. In ILD the relationship between weight status and disease outcomes is still unclear and may differ depending on the type of ILD. A previously mentioned, investigation by Alakhras et al. showed that a higher baseline BMI was associated with better survival in patients with IPF [23], while Kondoh et al. found that higher BMI in this IPF patient group was associated with increased risk of acute exacerbations [29]. To further complicate matters, Pugashetti et al. revealed that baseline BMI was not associated with survival, but included all subgroups of ILD in their study [30]. Therefore, it is unclear how obesity and high BMI impact disease outcomes and survival in ILD, and whether it is relates to the severity of illness or subtype of disease.

Handgrip strength is a strong indicator of muscle mass, muscle strength and functional status, especially in older adults [31]. More importantly, Peterson et al. found that HGS and respiratory muscle strength (expiratory, inspiratory and sniff nasal inspiratory pressure) were all strongly correlated [32]. Growing evidence suggests that muscle dysfunction, defined by the loss of strength and/or endurance [33], is prevalent in ILD [34] and involves both respiratory and peripheral muscles [35–37]. Muscle dysfunction has been linked to poorer health outcomes and reduced quality of life in both the healthy ageing population, and in chronic pulmonary disease population such as COPD [38, 39]. Similarly, studies have shown that for patients with IPF, the limitation in functional capacity caused by the disease is a strong driver of reduced quality of life in multiple domains. Other factors including symptoms and treatment side effects also influence quality of life.

The decline in muscle function in patients with ILD is probably multifactorial and is related to factors such as muscle disuse, hypoxaemia, malnutrition, oxidative stress, systemic inflammation and medications [40]. A reduction in physical activity seen in ILD patients is multifactorial, and may initially result from ventilatory limitation, as well as respiratory and psychological symptoms (cough, dyspnoea, anxiety and depression) which lead to exercise avoidance. This subsequently results in conditioning of different body components such as the cardiovascular system and limb muscles and bones, further limiting functional capacity [41]. Fortunately, the impairment in muscle function appears to be partially reversible with training [42]. Currently, optimal nutrition and early muscle reconditioning are considered to be the most effective strategies in reducing the impact of chronic lung diseases and low muscle strength on the quality of life of these individuals [43]. Pulmonary rehabilitation has been shown to improve exercise capacity, decrease dyspnoea and help to cope with daily activities in IPF patients, even though benefits are smaller and last for less time compared to other chronic lung diseases such as COPD [44, 45]. However, nutrition intervention at rehabilitation is not always a focus. It is known that inadequate protein intake to meet requirements results in compensatory losses of whole body protein, which is preferably lost from muscle mass leading to muscle atrophy and functional decline [46]. Thus, the inclusion of nutritional support in the multi-disciplinary ILD management may have a positive subsequent impact on quality of life through weight management and muscle loss prevention as supported by the correlation between quality of life and handgrip strength. Future research should include an assessment of nutritional interventions to provide specific support around weight management and malnutrition; and the impact on functional outcomes. This would inform service delivery models as to the allocation of allied health, particularly dietitians, within ILD multidisciplinary clinics.

Altered body composition in ILD, independent of weight status, is described in the literature. Results by Rinaldi et al. identified sarcopenic obesity in 10% of ILD patients based on high fat mass index and low fat-free mass index [47]. Interestingly, in the aforementioned study not all patients with sarcopenic obesity were identified as malnourished on SGA assessment. This suggests that nutritional status may be difficult to assess based on SGA and BMI parameters alone. In patients with advanced IPF awaiting lung transplantation, severe depletion of lean body mass, a circumstance generally associated with muscle dysfunction [41], was observed in as much as 56% of patients [48]. Although combined losses of body weight and lean mass were seen in some patients, lean body mass depletion was not always associated with low body weight as it was potentially masked by a relative abundance in fat mass. Moreover, a recent study has revealed a strong association between ILD severity and body composition. Individuals with a more impaired pulmonary function were found to have lower muscle mass and higher fat mass. Furthermore, men but not women in the study were found to have lower handgrip strength than expected compared to an age-matched healthy Canadian population [49].

More recent interest has focused on frailty as an index measure when assessing suitability for lung transplantation. Frailty is defined as decreased physiological reserve, and has been shown to be prevalent amongst ILD patients referred for a lung transplant and is associated with an increase in mortality [50]. Hence, greater exploration of the role of handgrip strength as an indicator of sarcopenia and/or frailty is required.

There are several limitations to this study. The cross-sectional study design allows only speculations about the causality of the associations between nutritional status and quality of life in patients with ILD. Moreover, we were unable to draw any solid conclusions regarding the impact of weight status on quality of life due to the low number of malnourished patients, hence, additional investigations are needed to confirm these findings. More detailed analysis of body composition using measures such as bioelectrical impedance or dual energy x-ray absorptiometry (DEXA) would also provide greater insight into changes in muscle and fat mass. Additionally, our regression models were only able to explain a small percentage of variance in quality of life, which suggests the important contribution of other factors that were not measured in this study. Lastly, EQ-5D health state index scores were estimated based on the existing value sets for the EQ-5D-3L questionnaire for the UK as value sets for Australia have not been published yet. The results of this study are not generalisable to all ILD patients as it was conducted in a specialised ILD clinic at a tertiary hospital. In order to improve generalisability, a multicentre study with a larger sample size should be conducted in the future which includes a wider range of patients with variable disease progression. However, this study is unique in that this cohort consisted of variable subtypes of ILD, as most previous research describing nutrition in ILD has been conducted in patients with IPF. Moving forward, studies measuring muscle mass, rather BMI alone, may provide better insight into muscle strength deterioration. Future research should aim to measure prevalence of sarcopenia using methods of body composition in this patient group as well as explore the role of obesity and decreased muscle strength on quality of life and functional outcomes including mortality.

## Conclusions

The present study indicates that mild-moderate malnutrition was only identified in a small number of patients with ILD. Unexpectedly, the vast majority of patients were found to be overweight and obese. The nutritional status of patients in different diagnostic groups did not appear to differ significantly. We also found that handgrip strength is compromised in this cohort and is linked to reduced quality of life. The association between handgrip strength and quality of life indicates the importance of nutrition interventions, alongside exercise programs for weight management and muscle loss prevention in this patient group. The results of this study support further research into the impact of obesity and decreased muscle strength on quality of life and health outcomes in patients with ILD.

## Data Availability

The datasets used and/or analysed during the current study are available from the corresponding author on reasonable request.

## Abbreviations

BMI: Body mass index
CF: Cystic fibrosis
COPD: Chronic obstructive pulmonary disease
CTD: Connective-tissue disease
DLCO: Diffusing capacity of the lungs for carbon monoxide
FFM: Fat free mass
FVC: Forced vital capacity
IPF: Idiopathic pulmonary fibrosis
ILD: Interstitial lung disease
K-BILD: King’s-Brief Interstitial-Lung-Disease
MAC: Mid-arm circumference
MAMC: Mid-arm muscle circumference
QoL: Quality of life
SGA: Subjective global assessment
TLC: Total lung capacity
TSF: Triceps skinfold
VAS: Visual Analogue Scale
